# Effects of supplementing rumen-protected rubber seed oil to dairy cattle on feed digestibility and milk production

**DOI:** 10.5713/ab.24.0287

**Published:** 2024-10-24

**Authors:** Pongsatorn Gunun, Norakamol Laorodphan, Warachit Phayom, Walailuck Kaewwongsa, Chatchai Kaewpila, Waroon Khota, Nirawan Gunun

**Affiliations:** 1 Department of Animal Science, Faculty of Natural Resources, Rajamangala University of Technology Isan, Sakon Nakhon 47160, Thailand; 2 Animal Science and Aquaculture Program, Faculty of Food and Agricultural Technology, Pibulsongkram Rajabhat University, Phitsanulok 65000, Thailand; 3 Energy Engineering, Faculty of Technology and Engineering, Udon Thani Rajabhat University, Udon Thani 41000, Thailand; 4 Department of Animal Science, Faculty of Technology and Engineering, Udon Thani Rajabhat University, Udon Thani 41000, Thailand

**Keywords:** Dairy Cows, Digestibility, Milk Composition, Milk Yield, Polyunsaturated Fatty Acids, Rumen-Protected Rubber Seed Oil

## Abstract

**Objective:**

The aim of this study was to determine the effect of rumen-protected rubber seed oil (RPRSO) supplementation on feed digestibility, milk yield, and milk composition in tropical dairy cows.

**Methods:**

Twelve crossbred Holstien-Friesian dairy cows (75% Holstein-Friesian, 25% Thai native breed) with a mean body weight of 460±30 kg and 20±5 days in milk were randomly assigned to 1 of 3 treatments according to a completely randomized design. The treatments were as follows: a basal diet without rumen-protected fat (RPF) (control) or supplementation of rumen-protected palm oil (RPPO) at 300 g/h/d and RPRSO at 300 g/h/d. Each cow was fed a total mixed ration *ad libitum*.

**Results:**

The nutrient intake was similar among treatments (p>0.05). Adding RPF did not affect nutrient digestibility, while organic matter (OM) digestibility increased in dairy cows receiving RPRSO (p<0.01). Blood urea nitrogen (BUN), total protein, or glucose did not alter among treatments (p>0.05), while triglycerides and cholesterol were increased when cows were fed RPPO (p<0.01). Adding RPF increases milk yield in cows (p<0.01). The supplementation of RPRSO increased milk fat (p = 0.04). Milk fat yield was higher in RPPO and highest in RPRSO (p<0.01). The addition of RPF increased the oleic acid (OA, C18:1 cis-9) in milk (p = 0.01). In addition, cows fed RPRSO increased linoleic acid (LA; C18:2ω6 cis-9,12+trans-9,12) and α-linolenic acid (ALA; C18-3ω3 cis-9,12,15) in milk (p<0.01). The addition of RPF increased milk unsaturated fatty acids and monounsaturated fatty acids (p≤0.04). The polyunsaturated fatty acid (PUFA) in milk increased with RPRSO supplementation (p<0.01).

**Conclusion:**

Supplementation of RPRSO during early lactation can increase feed digestibility and the concentration of milk fat with PUFA (LA and ALA) in tropical dairy cows.

## INTRODUCTION

The early lactation dairy cow is highly susceptible to experiencing a negative energy balance, which has an adverse impact on milk production [1]. Feeding rumen protected fat (RPF) provides more energy without affecting rumen functions, which may improve production and alleviate the negative effects of feeding low forage diets [2,3]. However, some studies have shown that adding RPF does not affect milk yield or composition [4]. Commercial products containing calcium salts produced from palm oil, which is rich in saturated fatty acids (SFA), are the most commonly recognized RPF supplements in ruminant.

Milk and dairy products are the main sources of SFA, which have been associated with an increased risk of cardiovascular disease (CVD) and cardiometabolic syndrome [5]. Currently, there is a growing trend among consumers toward an increased interest in healthy food products. Omega-3 (ω3) and omega-6 (ω6) polyunsaturated fatty acids (PUFA) have shown beneficial effects in CVD, chronic inflammation, and autoimmune disorders [6]. Linoleic acid (LA) and α-linolenic acid (ALA) have been considered essential fatty acids for humans [7]. However, the transfer efficiency of PUFA from the diet into milk in lactating cows is relatively low due to microorganisms from the rumen that are toxic to unsaturated fatty acids (UFA) because they damage cell integrity [8]. Therefore, following hydrolysis, rumen microorganisms convert UFA to SFA via a process known as biohydrogenation. The manipulation by using rumen-protected linseed or tuna oil has the potential to prevent rumen biohydrogenation and enhance the performance of milk fat with PUFA from ruminant [9,10].

The rubber tree (*Hevea brasiliensis*), primarily cultivated in Southeast Asia, produces rubber seed, a by-product consisting of shell and kernel. Due to its composition, which includes 40.0% LA and 21.2% ALA, the rubber seed could be used as a PUFA source [11]. In dairy cows, a previous study found that adding 4% rubber seed oil to the diet could manipulate the rumen fermentation pattern by increasing the proportion of propionate while reducing acetate and total volatile fatty acids [12]. Moreover, adding rubber seed oil increased milk yield and PUFA in milk, especially LA and ALA, while lowering milk fat and total solids in mid-lactating dairy cows [13]. Our hypothesis is that rumen-protected rubber seed oil (RPRSO) supplementation increases feed utilization, milk production, milk fat concentration, and the milk fatty acid profile. Therefore, the objective of this research was to evaluate the effect of supplementation with RPRSO on digestibility, milk yield, and milk composition in tropical dairy cows.

## MATERIALS AND METHODS

### Animal welfare

Animal care was approved by the Animals Ethical Committee of the Udon Thani Rajabhat University (approval number 0622.7/671 on September 19, 2022).

### Dietary preparation

From rubber orchards in Udon Thani, Thailand, fresh rubber seeds were harvested. After being hand-picked from the ground, the whole seed was housed indoors. Rubber seed oil is extracted by using an oil compression apparatus that operates based on a screw compression mechanism. Subsequently, the acquired oil should be subjected to filtration using No. 41 filter paper in order to obtain rubber seed oil that is suitable for the production of RPF. The preparation of RPRSO was produced using a method modified from that of Naik et al [14].

### Animals, experimental design, and treatments

This study was conducted at Racha dairy farm under the administration and supervision of the Dairy Farming Promotion Organization) in the northeastern region of Thailand. Twelve early-lactation crossbred dairy cows (75% Holstein-Friesian, 25% Thai native breed) were arranged in a completely randomized design pattern to receive three treatments. The cows with a mean body weight (BW) of 460±30 kg, 20±5 days in milk, and produced 18±1.0 kg of milk each day. Cows were fed a basal diet without RPF control) or supplemented with rumen-protected palm oil (RPPO) (commercial) at 300 g/h/d and RPRSO at 300 g/h/d. The cows were fed total mixed ration (TMR) (Table 1) *ad libitum* at 07:00 h and 16:00 h each day. Cows were milked twice daily at 06:00 and 15:00 h. Animals were utilized to assess performance during a 60-day period. The cows were kept in separate enclosures with access to mineral blocks and clean drinking water.

### Data collection and sampling procedures

The amount of feed offered and refused was recorded for each individual cow and taken for chemical analysis. Each animal's feces were collected to investigate digestibility during the final five days of the experiment (days 56 to 60). About 500 g of fresh fecal samples were obtained via rectal sampling in the morning (06:30 h) and afternoon (16:30 h). Two subsequent samples were combined, and the composite was subsequently kept in the refrigerator at 4°C. Feed, refusals, and feces samples were dried at 60°C and ground (1-millimeter screen) using the Cyclotech Mill (Tecator, Hoganas, Sweden). In the samples, the amounts of dry matter (DM) (method 930.15), ash (method 942.05), crude protein (CP) (method 955.04) [15], neutral detergent fiber (NDF), and acid detergent fiber (ADF) [16] were evaluated. To analyze feed fatty acid profiles using gas chromatography (GC 8890; Agilent Technologies Ltd., Santa Clara, CA, USA) [17] equipped with a capillary column (DB-WAX 30 m, 0.25 mm, 0.25 μm; Agilent Technologies Inc.) and flame ionization detector. The initial oven temperature was 140°C, held for 5 min, subsequently increased to 240°C at a rate of 4°C/min, and then held for 20 min. Helium was used as the carrier gas at a flow rate of 0.5 mL/min, and the column head pressure was 280 kPa. Both the injector and the detector were set at 260°C [18]. Nutritional digestibility was computed using measurements of the concentration of acid-insoluble ash (AIA) [19].

The amount of milk produced each day was recorded. Milk samples were also collected by milking machines during morning and afternoon milking and refrigerated at 4°C to determine fat, protein, lactose, total solids, and solids-not-fat by means of infrared methods using Milko-Scan 33 (Foss Electric, Hillerod, Denmark). The somatic cell count (SCC) was measured using a Fossomatic 5000 (Foss Electric). The fatty acid profile in milk was determined using gas chromatography (GC 8890; Agilent Technologies Ltd.) [17], which was similar to determining fatty acid in feed.

At 4 hours after feeding on the final day of the experiment, fresh blood samples were collected from the jugular vein of each cow in a volume of 10 mL. Each blood sample was kept in tubes containing ethylene diamine tetra acetic acid to assess the blood urea nitrogen (BUN), total protein, glucose, cholesterol, and triglyceride, which were measured with a chemical analyzer (Mindray BS-600; Mindray, Shenzhen, China).

### Statistical analysis

Data were analyzed using the MIXED procedure of SAS [20]. Milk yield was analyzed by repeated measurements using the following model:


$$Y_{ijk}=\mu +\alpha_{i}+\beta_{j}+{\left(\alpha \beta \right)}_{ij}+\delta_{ik}+{ɛ}_{ijk},$$

where *Y_ijk_* is the dependent variable, *μ* is the overall mean, *α_i_* is the fixed effect of treatment (*i* = 1 to 3), *β_j_* is the fixed effect of milk collection time (*j* = 1 to 4; the time of 0 to 15 d, 16 to 30 d, 31 to 45 d, and 46 to 60 d), (*αβ*)*_ij_* is the fixed interaction effect of *α_i_* and *β_j_*, *δ_ik_* is the random effect of the individual cow within αi, and ɛijk is the residual error. Time was prescribed as a repeated measure. The most appropriate covariance structure for repeated measures was selected based on the least Bayesian information criterion value. The least-squares-means were separated using the PDIFF option. Significant differences were accepted at p<0.05.

## RESULTS

### Chemical composition of diets

The DM and CP of TMR diets were 53.3% and 14.0% DM, respectively (Table 1). The RPPO and RPRSO contain 90.4% and 90.2% ether extract (EE), respectively. The contents of ALA, LA, and oleic acid (OA) in RPRSO were 11.24%, 40.98%, and 27.68%, respectively. Moreover, the RPRSO had a higher concentration of UFA and PUFA, especially LA and ALA. While RPPO contains higher SFA, especially palmitic acid (C16:0) (Table 2).

### Feed intake and nutrient digestibility

Supplementation with RPF did not influence the intake of DM, organic matter (OM), CP, NDF, or ADF (p≥0.05) (Table 3). The digestibility of DM, CP, NDF, or ADF was similar among treatments (p>0.05). While OM digestibility was increased in RPRSO (p<0.01).

### Blood chemistry

The inclusion of RPF did not affect the levels of BUN, total protein, or glucose (p>0.05) (Table 4). The addition of RPPO increased cholesterol and triglyceride concentrations (p<0.01).

### Milk production and compositions

Milk production was significantly increased when supplemented with RPPO and RPRSO (treatment, p<0.01). Milk fat was increased in cows fed RPRSO (p = 0.04) (Table 5). Milk fat yield was higher in RPPO and highest in RPRSO (p<0.01). However, milk protein, lactose, solids-not-fat, total solids, and SCC were similar among treatments (p>0.05).

### Fatty acid profile in milk

The addition of RPF increased butyric acid (C4:0) (p = 0.01) in milk (Table 6). Myristic acid (C14:0) and pentadecylic acid (C15:0) were reduced with the addition of RPPO (p = 0.04). Moreover, adding RPF enhanced tricosylic acid (C23:0) and OA (C18:1 cis-9) in milk (p≤0.03). The LA in milk was higher in RPRSO (p<0.01). Adding RPRSO increased the ALA in milk (p<0.01). RPF supplementation decreased milk SFA (p = 0.04), while increasing milk UFA, and MUFA (p≤0.04). The milk PUFA increased with the addition of RPRSO (p<0.01).

## DISCUSSION

The contents of OA, LA, and ALA in RPRSO were 27.68%, 40.98%, and 11.24%, respectively. Our previous studies found that heat treatment of rubber seed kernels had an amount of OA, LA, and ALA of 22.50%, 40.00%, and 21.20%, respectively [11]. The ALA content in the RPRSO was lower than in the previous report, which may be due to the different physical forms of rubber seed, the location of harvest, harvest period, and so on [21]. Another possibility was that rubber seed oil was the main ingredient to create RPF, along with calcium hydroxide, sulfuric acid, and butylated hydroxytoluene as minor ingredients, resulting in lower levels of ALA in RPRSO. In addition, Pi et al [12] reported that rubber seed oil contained UFA and PUFA and had contents of 83.00% and 59.00%, respectively. Similarly, our results found that RPRSO contained UFA and PUFA of 81.16 and 52.77, respectively. This suggests that ruminant diets could potentially use RPRSO as a feed additive.

Early lactation is characterized by a cow's lower feed intake and increased body condition score (BCS) mobilization in order to satisfy their milk requirement. Dairy cows use the BCS as an indicator of their energy balance status, and there is a correlation between the BCS, BW, and milk production [22]. In the present study, adding RPF did not influence dry matter intake (DMI), while increasing milk yield and milk fat in dairy cows. Similarly, Ranaweera et al [1] observed that tropical dairy cattle showed increased milk production, but supplementation with RPF had no effect on BW and DMI. Kowalski et al [23] reported that the addition of RPF had no effect on BW, BCS, or DMI in early lactation dairy cows. This may indicate that cows supplemented with RPF may have utilized fewer body reserves throughout the early lactation period, resulting in a maintained BW and BCS while providing energy for milk production and milk fat synthesis. However, the present experiment did not evaluate the BW and BCS of the cows, leaving the situation unclear.

Ruminants extensively use internal markers, especially AIA, to assess the nutrient digestibility from fecal samples. Fecal collection provides an accurate estimate of the diet's digestibility at any time during the day. Previous studies suggested spot sampling at least six times was accurate of fecal output in dairy cows [24]. Nevertheless, using fecal sampling twice a day for five days in our investigation may result in an estimation bias in the digestibility of nutrients. The digestibility of OM increased with the inclusion of RPRSO. The results agree with Ghoniem and Atia [25], who found that adding RPF to the diet of crossbred ewes increased the OM digestibility. However, previous studies showed that the addition of rubber seed oil did not affect OM digestibility in dairy cows [12]. RPF supplementation increased the digestibility of nutrients such as CP, EE, and crude fiber, which in turn increased the digestibility of OM in ruminants [21]. In the current study, this might increase the digestibility of CP, NDF, and ADF of RPRSO, despite their non-significant differences, and also lead to an increase in OM digestibility in dairy cows.

The diets had a significant effect on the content of triglycerides and total cholesterol in the cows’ blood serum. Animals with high triglycerides often have a high total cholesterol level [26]. Our results demonstrate the relationship between triglyceride and cholesterol levels. The concentrations of cholesterol and triglycerides were increased in cows fed RPPO, as found in the present study. Others have also observed that the concentrations of cholesterol and triglycerides in the blood will increase in cows fed RPPO in buffalo [27]. These results may be attributed to triglyceride-rich lipoproteins being secreted into the lymphatic system and eventually released into the main blood circulation via the thoracic duct [13]. However, supplementation with RPRSO can lower levels of triglycerides and cholesterol compared to RPPO. Clearly, the level of cholesterol in the milk is closely dependent on the quantity of fat matter.

Feeding of RPF had the maximum effect on milk yield during the first quarter of lactation, when feed intake is usually low, and the effect was less prominent as lactation advanced, probably due to the DM intake starting to increase after 6–8 weeks of calving [28]. The increased milk yield observed in the RPF group may be attributed to the increased energy density of the ration, resulting in reducing the deleterious effect of negative energy balance [3]. Previous studies reported that adding RPF could increase milk yield in tropical crossbred dairy cows [1]. In the current study, adding RPF increased milk yield in lactating cows when compared with the control group. However, it was not affected by time or the interaction of treatment×time effect. There are two plausible reasons. First, the difference in milk yield was not caused by the treatments but rather by the different groups of cows that were selected and assigned to different treatments. Hence, the difference was maintained throughout the experiment, resulting in effects that were not affected by time and interaction effects but by treatment effects. Secondly, the treatments immediately altered the milk production once the treatment diets were fed from the first week of the experiment. Then, it could have a significant treatment effect without any effect of time or interaction. These findings are consistent with those of Price et al [29], who found that Holstein dairy cows increased milk production when changing the treatment diets in the immediate period (days 3 and 4) and short-term adaptation period (days 7 and 8). Nevertheless, it is very difficult to determine milk production adjustments during the initial week, as animals require a minimum of two weeks to substitute gut digesta with treatment diets.

Milk fatty acid is synthesized either from fatty acids taken up from the blood or by de novo synthesis in the mammary gland. In addition, among all components of milk fat content, it is most sensitive to dietary changes. The addition of RPRSO to a diet generally increases the milk fat percentage and milk fat yield due to an increase in fatty acid uptake. There are two plausible explanations for this result. First, adding RPRSO, which contains major long-chain fatty acids, will increase the milk fat content. Second, the use of RPRSO may be non-toxic to microbes, especially cellulolytic bacteria, and have no negative effect on fiber intake and digestibility when compared to the control, which ultimately results in increased milk fat.

The rubber seed oil by-products from tropical rubber plantations contain a high level of OA, LA, and ALA, which makes them viable sources of UFA for animals [30]. However, the biohydrogenation may change LA and ALA to C18:0 in the rumen when ruminants are fed C18 UFA in their diets. The inhibited biohydrogenation of C18 UFA in rubber seed has been found in our previous studies. Gunun et al [11] found that the inclusion of rubber seed kernel heated in a hot air oven reduced biohydrogenation in the rumen and increased levels of OA, LA, and ALA *in vitro*. So, a study using rumen-protected UFA might enhance milk fat with PUFA and also be used for functional foods. In the current study, the addition of RPPO and RPRSO resulted in an increase in OA compared to the control group. The contents of OA in RPPO and RPRSO were 37.16% and 27.68%, respectively. When dietary RPF supplementation in cows leads to increased OA in milk. Furthermore, adding RPRSO increased the LA and ALA in milk. Similarly, Pi et al [13] found that adding 4% rubber seed oil increased OA, LA, and ALA in early lactation dairy cows. The levels of LA and ALA in milk increased in cows fed RPRSO (2.8% and 0.38%, respectively) when compared to the control (1.5% and 0.14%, respectively), which correlated with higher concentrations of LA and ALA in these diets. This could be due to the fact that using RPF has the potential to protect UFA against ruminal dissociation. As a result, there is an increased supply of UFA in the small intestine, allowing it to be available to the mammary gland for incorporation into milk fat with a higher LA and ALA content [31].

Milk fat synthesis is nutritionally modulated to increase the concentration of PUFA [32]. According to Pi et al [13], adding rubber seed oil increases PUFA in milk, whereas reducing milk fat in dairy cows may affect biohydrogenation in the rumen. The strategies to use rumen-protected oil lead to an enhanced transfer of PUFA to the small intestines while having no effect on rumen fermentation or milk fat concentration [33]. In the present study, adding RPRSO increased milk PUFA and also raised milk fat in dairy cows. These findings are in agreement with Contreras-Solís et al [9], who found that supplementation with rumen-protected linseed oil in sheep increases milk PUFA. This indicated that RPRSO enabled intestinal absorption in the gut and tissue distribution, which includes the udder [32], and also enhanced milk PUFA in early lactation dairy cows. However, this study used a small sample size for dairy cattle (n = 4 per treatment), probably showing the weak point. The impact of this investigation on the digestibility and performance of cows may be greater if the use of RPF was consistent with a large sample size.

## CONCLUSION

The RPRSO supplementation increases OM digestibility. Cows fed RPPO had the highest concentrations of blood cholesterol and triglycerides. Moreover, adding RPRSO increases milk fat concentration and PUFA, particularly LA and ALA, in milk. Therefore, supplementing RPRSO during early lactation enhances feed digestibility and milk fat with PUFA in tropical dairy cows.

## Figures and Tables

**Table 1:** Ingredient and chemical composition of experimental diet

Item	TMR	RPPO	RPRSO
Ingredient (% DM)			
Corn silage	60.0		
Rice straw	17.0		
Soybean meal	18.5		
Cassava pulp	2.5		
Vitamins^1)^	1.5		
Minerals^2)^	0.5		
Chemical composition			
Dry matter (%)	53.3	98.9	99.0
Organic matter (%DM)	84.1	78.4	75.1
Crude protein (%DM)	14.0	-	-
Ether extract (%DM)	1.8	90.4	90.2
Neutral detergent fiber (%DM)	58.8	-	-
Acid detergent fiber (%DM)	22.6	-	-

TMR, total mixed ration; RPPO, rumen-protected palm oil; RPRSO, rumen-protected rubber seed oil.

1) Contains per kilogram vitamin: 5,000,000 IU vitamin A; 1,000,000 IU vitamin D; 11,000 IU vitamin E.

2) Contains per kilogram mineral: 107.78 g Na; 93.72 g Ca; 46.86 g P; 18.55 g S; 8.24 g Mn; 7.49 g Zn; 3.37 g Mg; 1.17 g Cu; 0.15 g Co; 0.04 I; 0.02 Se; 0.01 g K.

**Table 2:** Fatty acid profile of rumen-protected palm oil (RPPO) and rumen-protected rubber seed oil (RPRSO)

Fatty acid (% of total fatty acid)	RPPO	RPRSO
C6:0	0.07	0.05
C8:0	0.02	0.01
C10:0	0.01	0.02
C12:0	0.17	0.01
C14:0	1.04	0.10
C15:0	0.05	0.01
C16:0	50.53	10.54
C16:1 cis-9	0.12	0.20
C17:0	0.09	0.02
C18:0	3.91	7.69
C18:1 cis-9 (OA)	37.16	27.68
C18:2 cis-9,12+trans-9,12 (LA)	5.75	40.98
C18:3 cis-9,12,15 (ALA)	0.18	11.24
C20:0	0.23	0.27
C20:1 cis-11	0.39	0.09
C20:5 cis-5,8,11,14,17	0.06	0.55
C22:0	0.03	0.06
C22:1 cis-13	0.06	0.37
C22:6	0.01	0.04
C23:0	0.11	0.07
SFA	56.27	18.84
UFA	43.73	81.16
MUFA	37.74	28.38
PUFA	5.99	52.77

OA, oleic acid; LA, linoleic acid; ALA, α-linolenic acid; SFA, saturated fatty acids; UFA, unsaturated fatty acids; MUFA, monounsaturated fatty acids; PUFA, polyunsaturated fatty acids.

**Table 3:** Effect of supplementation of rumen-protected palm oil (RPPO) and rumen-protected rubber seed oil (RPRSO) on nutrient intake and digestibility in dairy cows

Item	Control	RPPO	RPRSO	SEM	p-value
Nutrient intake (kg/d)					
Dry matter	14.4	14.3	14.0	0.17	0.29
Organic matter	12.1	12.1	11.8	0.14	0.30
Crude protein	2.0	2.0	2.0	0.14	0.85
Neutral detergent fiber	8.5	8.4	8.4	0.08	0.05
Acid detergent fiber	4.6	4.5	4.5	0.05	0.64
Digestibility coefficients (%)					
Dry matter	50.3	54.6	60.0	3.28	0.18
Organic matter	51.0^a^	57.8^ab^	61.8^b^	2.09	<0.01
Crude protein	59.2	61.9	66.3	2.50	0.19
Neutral detergent fiber	52.1	52.1	59.4	3.13	0.20
Acid detergent fiber	40.1	42.9	47.8	4.35	0.48

SEM, standard error of the mean.

a,b Values in the same row with different superscripts differ according to p<0.01.

**Table 4:** Effect of supplementation of rumen-protected palm oil (RPPO) and rumen-protected rubber seed oil (RPRSO) on blood chemistry in dairy cows

Item	Control	RPPO	RPRSO	SEM	p-value
BUN (mg/dL)	14.5	13.0	12.3	1.20	0.44
Total protein (g/dL)	6.6	6.6	6.7	0.23	0.84
Glucose (mg/dL)	55.0	53.1	53.0	1.73	0.15
Cholesterol (mg/dL)	141.5^a^	200.7^b^	164.7^a^	14.26	<0.01
Triglyceride (mg/dL)	6.3^a^	10.3^b^	6.0^a^	0.61	<0.01

SEM, standard error of the mean; BUN, blood urea nitrogen.

a,b Values in the same row with different superscripts differ according to p<0.01.

**Table 5:** Effect of supplementation of rumen-protected palm oil (RPPO) and rumen-protected rubber seed oil (RPRSO) on milk yield and milk composition in dairy cows

Item	Control	RPPO	RPRSO	SEM	p-value		

					Trt	Time	Trt×Time
Production (kg/d)							
Milk yield	17.9^a^	21.4^b^	21.0^b^	0.62	<0.01	0.09	0.99
4% FCM	17.0	19.9	20.9	1.93	0.18	0.28	0.99
Milk composition (%)							
Fat	3.4^a^	3.6^a^	4.0^b^	0.09	0.04	-	-
Protein	3.3	3.4	3.4	0.06	0.48	-	-
Lactose	4.7	4.4	4.5	0.07	0.13	-	-
Solids-not-fat	8.3	8.1	8.2	0.05	0.08	-	-
Total solids	12.4	12.7	12.8	0.10	0.12	-	-
Milk composition yield (kg/d)							
Fat	0.41^a^	0.46^b^	0.51^c^	0.01	<0.01	-	-
Protein	0.4	0.4	0.4	0.01	0.35	-	-
Lactose	0.6	0.6	0.6	0.01	0.61	-	-
Solids-not-fat	1.0	1.0	1.0	0.06	1.00	-	-
Total solids	1.5	1.6	1.6	0.03	0.22	-	-
Somatic cell counts (cell/mL)10^5^	2.5	3.4	2.4	0.72	0.91	-	-

SEM, standard error of the mean; Trt, treatment; FCM, fat-collected milk.

a–c Values in the same row with different superscripts differ according to p<0.05 and p<0.01.

**Table 6:** Effect of supplementation of rumen-protected palm oil (RPPO) and rumen-protected rubber seed oil (RPRSO) on fatty acid profile in milk of dairy cows

Fatty acid (% of total fatty acid)	Control	RPPO	RPRSO	SEM	p-value
C4:0	0.8^a^	1.0^b^	1.0^b^	0.05	0.01
C6:0	0.8	0.8	0.9	0.06	0.62
C8:0	0.7	0.6	0.7	0.06	0.51
C10:0	2.1	1.6	2.0	0.23	0.29
C11:0	0.02	0.02	0.02	0.004	0.43
C12:0	2.6	1.8	2.3	0.27	0.15
C13:0	0.06	0.04	0.05	0.007	0.17
C14:0	13.5^a^	10.3^b^	12.6^ab^	0.79	0.04
C15:0	0.9^a^	0.6^b^	0.8^ab^	0.07	0.04
C16:0	44.7	41.8	39.2	1.58	0.10
C17:0	0.4	0.3	0.4	0.06	0.25
C18:0	8.1	10.2	8.3	1.25	0.47
C20:0	0.09	0.10	0.09	0.01	0.78
C22:0	0.03	0.03	0.03	0.008	0.90
C23:0	0.09^a^	0.16^b^	0.18^b^	0.02	0.03
C14:1 cis-9	0.9	0.5	0.9	0.19	0.36
C16:1 cis-9	1.6	1.4	1.6	0.22	0.81
C18:1 cis-9 (OA)	20.8^a^	26.3^b^	25.5^b^	1.07	0.01
C18:2 cis-9,12+trans-9,12 (LA)	1.5^a^	2.0^a^	2.8^b^	0.17	<0.01
C18:3cis-9,12,15 (ALA)	0.14^a^	0.14^a^	0.38^b^	0.04	<0.01
C20:1 cis-11	0.07	0.08	0.10	0.02	0.64
C20:5 cis-5,8,11,14,17	0.03	0.03	0.05	0.006	0.19
SFA	74.9^a^	69.4^b^	68.6^b^	0.85	0.04
UFA	25.1^a^	30.5^ab^	31.4^b^	1.20	0.03
MUFA	23.3^a^	28.3^b^	28.1^b^	0.82	0.04
PUFA	1.7^a^	2.2^b^	3.2^c^	0.21	<0.01

SEM, standard error of the mean; OA, oleic acid; LA, linoleic acid; ALA, α-linolenic acid; SFA, saturated fatty acids; UFA, unsaturated fatty acids; MUFA, monounsaturated fatty acids; PUFA, polyunsaturated fatty acids.

a–c Values in the same row with different superscripts differ according to p<0.05 and p<0.01.
